# Pain Management in Acute Pancreatitis: A Systematic Review and Meta-Analysis of Randomised Controlled Trials

**DOI:** 10.3389/fmed.2021.782151

**Published:** 2021-12-17

**Authors:** Wenhao Cai, Fei Liu, Yongjian Wen, Chenxia Han, Manya Prasad, Qing Xia, Vikesh K. Singh, Robert Sutton, Wei Huang

**Affiliations:** ^1^Department of Integrated Traditional Chinese and Western Medicine, Sichuan Provincial Pancreatitis Centre and West China-Liverpool Biomedical Research Centre, West China Hospital, Sichuan University, Chengdu, China; ^2^Liverpool Pancreatitis Research Group, Liverpool University Hospitals NHS Foundation Trust and Institute of Systems, Molecular and Integrative Biology, University of Liverpool, Liverpool, United Kingdom; ^3^Department of Anaesthesiology, Laboratory of Anaesthesia and Critical Care Medicine, National-Local Joint Engineering Research Centre of Translational Medicine of Anaesthesiology, West China Hospital, Sichuan University, Chengdu, China; ^4^Clinical Research and Epidemiology, Institute of Liver and Biliary Sciences, New Delhi, India; ^5^Division of Gastroenterology, Department of Medicine, Johns Hopkins Medical Institutions, Baltimore, MD, United States

**Keywords:** pain management, acute pancreatitis, analgesics, randomised-controlled trial, meta-analysis

## Abstract

**Background:** Pain management is an important priority in the treatment of acute pancreatitis (AP). Current evidence and guideline recommendations are inconsistent on the most effective analgesic protocol. This systematic review and meta-analysis of randomised controlled trials (RCTs) aimed to compare the safety and efficacy of analgesics for pain relief in AP.

**Methods:** A literature search was performed to identify all RCTs assessing analgesics in patients with AP. The primary outcome was the number of participants who needed rescue analgesia. Study quality was assessed using Jadad score. Pooled odds ratios (ORs) or weighted mean differences (WMDs) with 95% confidence intervals (CI) were analysed using a random-effects model.

**Results:** Twelve studies comprising 699 patients with AP (83% mild AP) were analysed. The tested analgesics significantly decreased the need for rescue analgesia (3 studies, OR.36, 95% CI 0.21 to 0.60) vs. placebo or conventional treatment. The analgesics also improved the pain score [Visual Analogue Scale (Δ-VAS)] at 24 h (WMD 18.46, 0.84 to 36.07) and by the 3rd to 7th days (WMD 11.57, 0.87 to 22.28). Opioids vs. non-opioids were associated with a decrease in the need for rescue analgesia (6 studies, OR 0.25, 95% CI 0.07 to 0.86, *p* = 0.03) but without significance in pain score. In subgroup analyses, opioids were similar to non-steroidal anti-inflammatory drugs (NSAIDs) regarding the primary outcome (4 studies, OR 0.56, 95% CI 0.24 to 1.32, *p* = 0.18). There were no significant differences in other clinical outcomes and rate of adverse events. Other studies, comparing epidural anaesthesia vs. patient-controlled analgesia and opioid (buprenorphine) vs. opioid (pethidine) did not show significant difference in primary outcome. Study quality issues significantly contributed to overall study heterogeneity.

**Conclusions:** NSAIDs and opioids are equally effective in decreasing the need for rescue analgesia in patients with mild AP. The relative paucity of trials and high-quality data in this setting is notable and the optimal analgesic strategy for patients with moderately severe and severe AP still requires to be determined.

## Introduction

Pain is one of the most prevalent and costly health conditions. The estimated cost of pain in the United States was more than that of heart disease and cancer treatments, reaching $560 billion to $635 billion per year (1). Acute abdominal pain is the leading symptom and principal reason for hospital admission in patients with acute pancreatitis (AP).

Acute pancreatitis (AP) is one of the most common gastrointestinal diseases with an increasing global incidence (2), for which there is no specific targeted therapy (3). According to the latest epidemiological investigation, aggregate annual health care cost of AP rose to $2.6 billion in the United States in 2014 (4) and £200 million in the United Kingdom (NHS). Abdominal pain not only serves as one of the diagnostic criteria (5), but also a prognostic factor (6), and is related to the length of hospital stay and other self-reported outcomes of patients with AP (7, 8).

Nearly all patients with AP experienced abdominal pain which warrants prompt analgesia, and this is one of the main management priorities in the early management of AP (9). Some current practise guidelines have overlooked recommendations for pain management (10–14), while others provide a clear recommendation for pain assessment (15, 16), and best pharmacological options (15) (Table 1). No guidelines provide sufficient details regarding the type, dose, route, and frequency of analgesia administration.

**Table 1:** Acute Pancreatitis (AP) guideline recommendations for pain management.

**AP guidelines**	**Recommendations for pain management**
2013 IAP/APA Evidence-based Guidelines for the Management of Acute Pancreatitis	No recommendations
2013 American College of Gastroenterology Guideline: Management of Acute Pancreatitis	No recommendations
2015 Japanese Guidelines for the Management of Acute Pancreatitis	Recommendation: Pain associated with AP is severe and persistent, raising the need of sufficient pain control. (Strong recommendation, high-quality evidence)
2015 The Italian Association for the Study of the Pancreas: Consensus guidelines on severe acute pancreatitis	No recommendations
2018 NICE guideline: pancreatitis	No recommendations for pain management in acute pancreatitis
2018 American Gastroenterological Association Institute Guideline on Initial Management of Acute Pancreatitis	No recommendations
2019 WSES guidelines for the management of severe acute pancreatitis	Recommendation: No evidence or recommendation about any restriction in pain medication is available. NSAIDs should be avoided in acute kidney injury. Epidural analgesia should be an alternative or an agonist with intravenous analgesia, in a multimodal approach. Patient-controlled analgesia should be integrated with every described strategy. (Strong recommendation, low-quality evidence)

*AP, acute pancreatitis; IAP, International Association of Pancreatology; APA, American Pancreatic Association; NICE, National Institute for Health and Care Excellence; WSES, World Society of Emergency Surgery; NSAID, non-steroidal anti-inflammatory drug*.

There are many pharmacological options, with opioids being the most frequently prescribed analgesics for pain relief of patients with AP. Since abdominal pain in AP is secondary to pancreatic parenchymal inflammation (17, 18), non-steroidal anti-inflammatory drugs (NSAIDs) that target the enzyme cyclooxygenase (COX) are often used (19, 20). Much less frequently, local anaesthetics (i.e., procaine and bupivacaine) and paracetamol (19–21) are used.

A previous meta-analysis of only 5 studies (22) (227 participants) showed there was no significant difference between opioids vs. non-opioids (3 studies, 162 participants) regarding the need for rescue analgesia, pain intensity, clinical outcomes, and adverse events for pain relief of patients with AP. It is noted that opioid requirement during hospitalisation is strongly associated with more complications of this disease (23, 24) and there is the need for more evidence regarding the safety and efficacy of individual opioids in AP management. Further, a recent systematic review (25) demonstrated that NSAIDs reduced AP severity and mortality rate in animal models and patients. However, this review included studies that were of poor quality, the efficacy of NSAIDs for pain relief in this setting still needs to be addressed. Recently, thoracic epidural anaesthesia has been shown to significantly improve pancreatic microcirculation and splanchnic perfusion and reduce indices for multiple organ failure and mortality (26). There is the need to repeat a systematic review on this topic and it includes recent clinical studies of pain relief protocols in patients with AP to guide clinical management and trials.

The aim of this systematic review and meta-analysis was to include recent randomised clinical trials (RCTs) to evaluate the safety and efficacy of analgesics in the management of pain in patients with AP.

## Methods

The study was conducted following protocols of the Preferred Reporting Items for Systematic reviews and Meta-Analyses (PRISMA) Statement (27).

### Literature Search Strategy

A thorough literature search was carried out in the databases of Ovid Medline (PubMed), EMBASE, Science Citation Index Expanded, and Cochrane Library up to June 23, 2021. The detailed search strategy is shown in Appendix 1 (supplementary material). All RCTs investigating pain management in AP were collated. Reference lists of relevant reviews and other non-primary data sources regarding this context captured by the search strategy were also manually screened. Only publications in English were included. Relevant articles were manually reviewed by three investigators (W.C., F.L., and Y.W.).

### Study Selection

Inclusion criteria for eligible studies were: (1) RCTs carried out in patients with AP; (2) treatment with opioids, NSAIDs, systemic, or epidural administration of local anaesthetics for abdominal pain; and (3) include at least one outcome measure for analysis. Non-RCTs, retrospective studies, reviews, abstracts, case reports/series, editorials, expert opinions, and non-English publications were excluded.

### Outcomes of Interest

The primary outcome was the number of patients requiring rescue analgesia beyond the analgesic being tested during the observation time of the trial (thereafter called “need for rescue analgesia”) (28). It is provided that a patient requesting rescue analgesic for multiple times is not counted more than once. This was chosen as the primary outcome because this was widely used in the trials, and it was considered more objective than the multiple patient-reported pain scoring systems. The rescue analgesia could be the same analgesic in the trial arm or a different analgesic.

Secondary outcomes included change of pain score, all complications, mortality, length of hospital stay, and adverse event rate. Pain scores were converted to a 0–100 scale Visual Analogue Scale (VAS) from the 0–5 or 0–10 scales for pooled calculation, and their change (Δ-VAS) was assessed within 24 h, at 72 h, and/or on 3–7 d after randomisation. Any complication was defined as development of local complication (acute peripancreatic fluid collection, acute necrotic collection, walled-off necrosis, pancreatic pseudocyst, and vein thrombosis) (5), organ failure (acute respiratory failure, acute liver injury, acute kidney injury, and cardiovascular failure), and other complications (e.g., altered bowel function, ileus, ascites, and pleural effusion) that were not attributed to an adverse drug reaction.

### Data Collection

Two authors (W.C. and Y.W.) independently extracted data from included studies using a standardised *pro forma* designed by a senior author (W.H.). These included: authors, year of publication, country, centre(s), sample size calculation, number of patients screened (analysed), patient baseline characteristics (age, gender, weight or body mass index, aetiology, disease severity), trial criteria, and process (time of pain to admission, inclusion and exclusion criteria, treatment details of all comparison groups, observation duration, assessment tool and frequency of pain, pain scores, frequency and dose of rescue analgesic, and outcome measures).

### Study Quality and Evidence Quality Assessment

Two authors (W.C. and Y.W.) scored each included study by using the Jadad system (29) that assesses randomisation (0 or 1), double blinding (0, 1, or 2), recording of dropouts and/or withdrawals (0 or 1), and allocation concealment (0 or 1), with a score ≥ 3 indicative of high quality.

The Grading of Recommendations, Assessment Development and Evaluations (GRADE) approach was used to assess the quality of the supporting evidence for selected outcomes using the GRADEpro software (https://gdt.gradepro.org). The quality of the evidence was classified as high, moderate, low, or very low based on consideration of the risk of bias, the directness of evidence, consistency, and precision of the estimates.

### Statistical Analysis

Quantitative data were pooled by meta-analyses using RevMan 5.3.5 (Cochrane Collaboration; Oxford, UK). For dichotomous variables, pooled effect estimates were calculated using odds ratios (ORs) with 95% confidence intervals (c.i.). Means and SDs of continuous variables were used for meta-analysis and estimated when medians with ranges were given to generate weighted mean difference (WMD) with 95% c.i. (30). All probability (*P*) values were two-tailed, and a *p* < 0.05 was considered statistically significant. Heterogeneity was evaluated by Cochrane's *Q* statistic with *P* value. *I*^2^ values were used to quantify the degree of heterogeneity (*I*^2^ ≥ 50% or *p* < 0.1 indicative of high heterogeneity). A random effect model was employed to pool the overall effect estimate. Subgroup analyses of the primary outcome were conducted by separately analysing studies comparing the same type of analgesics. Sensitivity analysis of primary outcome was performed by restricting studies with high quality (Jadad ≥ 3), sample size ≥ 40, Western population, and by studies mainly including patients with mild AP.

Statistical analysis of publication bias was performed using StatsDirect 3.0 (StatsDirect Ltd; Birkenhead, UK), and *p* values were generated from Begg-Mazumdar (31) and Egger (32) tests as *p* < 0.10 was also considered significant.

## Results

### Design and Quality Assessment of Included Studies

**Figure 1:**
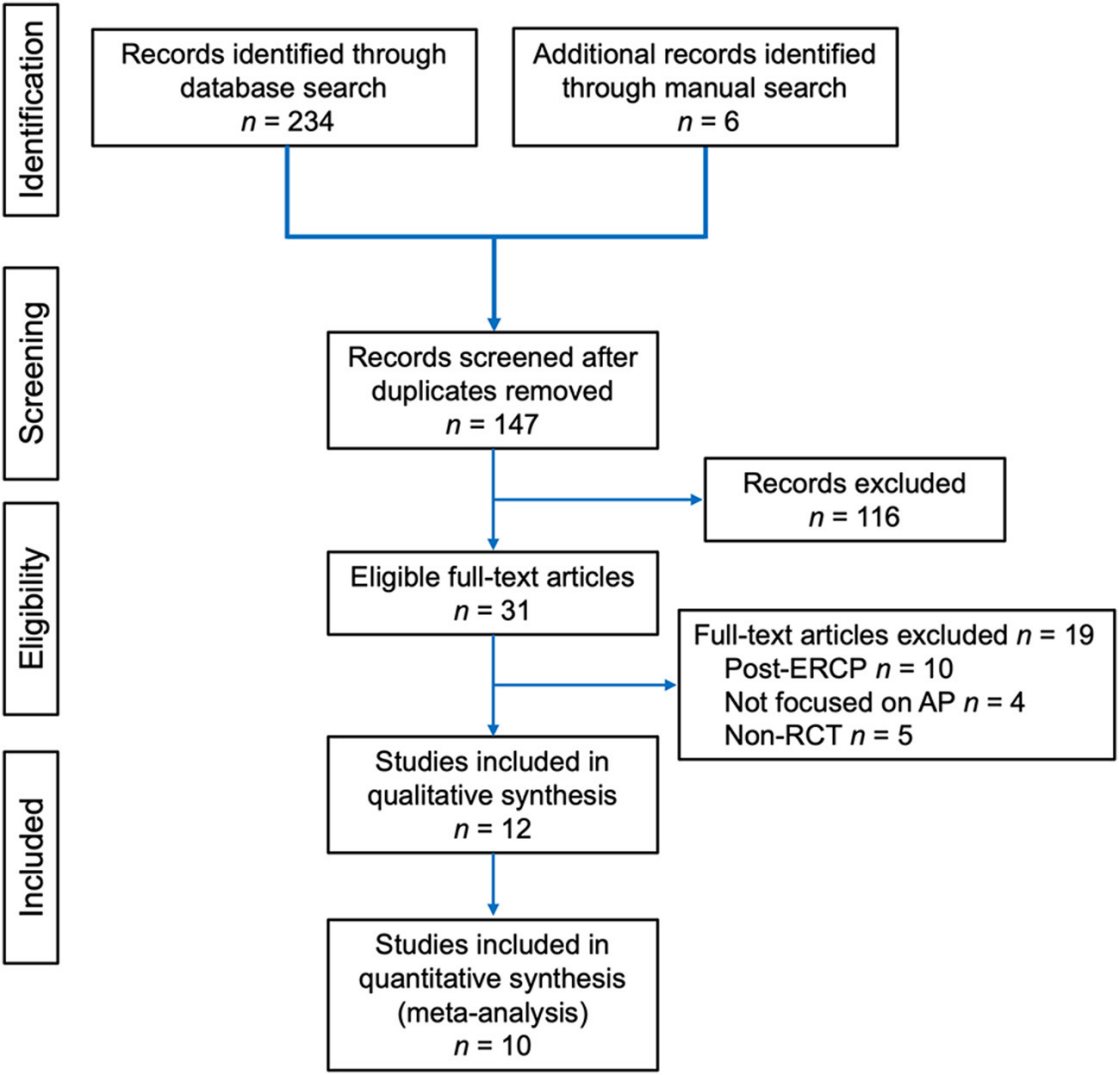
Preferred Reporting Items for Systematic reviews and Meta-Analyses (PRISMA) flow diagram of article selection for the review.

A PRISMA flow diagram for study selection is shown in Figure 1. A total of 12 studies (33–44) were included. Study design and quality assessment are displayed in Table 2 and a detailed Jadad scoring is shown in Supplementary Table 1. Only one study (36) was conducted in multiple centres. Three studies (36, 37, 41) performed sample size calculation. Seven (33, 35, 36, 38, 41, 42, 44) and 5 studies (34, 37, 39, 40, 43) had Jadad score ≥ 3 and ≤ 2, respectively.

**Table 2:** Design and quality assessment of included studies.

**Refrences**	**Country**	**Comparison groups**	**Centre**	**Patient type**	**Sample size calculation**	**Jadad score**
Blamey et al. (33)	UK	Opioid (buprenorphine) vs. Opioid (pethidine)	Single	Consecutive	No	3
Ebbehoj et al. (34)	Denmark	NSAID (indomethacin) vs. Placebo	Single	Consecutive	No	2
Jakobs et al. (39)	Germany	Opioid (buprenorphine) vs. local anaesthetic (procaine, i.v.)	Single	Consecutive	No	2
Stevens et al. (44)	USA	Opioid (TTS fentanyl) vs. Placebo	Singe	Consecutive	No	5
Kahl et al. (40)	Germany	Opioid (pentazocine) vs. local anaesthetic (procaine, i.v.)	Singe	Non-consecutive	No	2
Peiró et al. (43)	Spain	Opioid (morphine) vs. NSAID (metamizole)	Single	Non-consecutive	No	2
Layer et al. (36)	Germany	Local anaesthetic (procaine, i.v.) vs. Placebo	Multiple	Consecutive	Yes, difference of 20 VAS and SD of 24 points	4
Sadowski et al. (37)	Switzerland	Local anaesthetic (bupivacaine, PCEA) + Opioid (fentanyl, PCEA) vs. Opioid (fentanyl, PCIA)	Single	Non-consecutive	Yes, to detect an OR of > 2.5 with a power of 80% (*p* < 0.05), 50 patients are needed	2
Gülen et al. (38)	Turkey	Opioid (tramadol) vs. NSAID (paracetamol) vs. NSAID (dexketoprofen)	Single	Consecutive	No	4
Mahapatra et al. (42)	India	Opioid (pentazocine) vs. NSAID (diclofenac)	Single	Non-Consecutive	No	4
Huang et al. (35)	China	NSAIDs (parecoxib + celecoxib) vs. Conventional treatment	Single	Consecutive	No	3
Kumar et al. (41)	India	Opioid (tramadol) vs. NSAID (diclofenac)	Single	Consecutive	Yes, difference of SD of 30 mm for VAS, considering 30% dropout, 46 patients are needed	5

*NR, not reported; NSAID, non-steroidal anti-inflammatory drugs; TTS, transdermal therapeutic system; VAS, visual analogue scale; SD, standard deviation; PCEA, patient-controlled epidural anaesthesia; PCIA, patient-controlled intravenous analgesia; OR, odds ratio*.

### Baseline Characteristics of Included Patients

These data are shown in Supplementary Table 2. A total of 800 patients with AP were screened and 699 were analysed with age ranged from 23 to 86 years old. Gender was reported in 11 studies (34–44). Two studies (39, 40) reported the weight and 3 studies (35–37) reported body mass index. Ten studies (34–43) reported aetiology of AP. Regarding AP severity classification, 2 studies (38, 44) did not report data on AP severity, 2 studies (35, 37) reported substantial proportion of patients with severe AP, and the remaining 8 studies (33, 34, 36, 39–43) mainly included patients with mild AP. Pooled prevalence of mild AP was estimated to be 83% (478/577).

### Details of Trials

These are listed in Table 3. Ten studies (33–41, 43) were used the VAS to assess pain intensity, with VAS (0–100) in 6 studies (36, 38–41, 43) and VAS (0–10) in 4 studies (33–35, 37). One study (44) employed verbal self-report scale (0–5) for pain assessment and one (42) did not score pain. Study duration ranged from.5 h to 7 days.

**Table 3:** Details of trial criteria and process.

**Study**	**Sample size**	**Time of pain to admission**	**Inclusion and exclusion criteria**	**Intervention group**	**Control group**	**Observation time**	**Pain assessment (tool and frequency)**	**Rescue analgesia**	**Outcome measures**
Blamey et al. (33)	32	NR	NR	Pethidine (100 mg, i.m.)	Buprenorphine (0.3 mg, i.m.)	24 h	VAS (0–10); baseline and 24 h after treatment	Pethidine (100 mg, i.m.)	VAS (0–10)
Ebbehoj et al. (34)	30	NR	NR	Indomethacin (50 mg, rectal, twice/day)	Placebo	168 h	VAS (0–10); daily	Opiates	VAS (0–10); pain free days; number of rescue analgesic injections during 7 days treatment
Jakobs et al. (39)	40	NR	*Included:* Not specified; *Excluded:* Cardiac arrhythmias or previous severe arrhythmias, pregnancy, contradictions to anaesthetics	Procaine (2 g/day, i.v.)	Buprenorphine (0.3 mg bolus maintained with 2.4 mg per day, i.v.)	72 h	VAS (0–100); 3 times a day	Opiates	VAS (0–100); need for rescue analgesia
Stevens et al. (44)	32	NR	*Included:* Pain ≥ 2 on verbal self-report scale (0–5); *Excluded:* Respiratory diseases, contradictions to anaesthetics	Fentanyl (50 mg/h, TTS for 3 days)	Placebo	72 h or discharged earlier	McGill-Melzack Pain Q and verbal self-report scale (0–5); every 3 h	Demerol (50–100 mg, every 3 h as needed for 3 days)	Verbal self-report scale (0–5); total amount of fentanyl and Demerol
Kahl et al. (40)	101	<72 h	*Included:* Not specified; *Excluded:* Any analgesic prior to hospitalisation and pregnancy	Pentazocine (30 mg/kg, i.v., every 6 h)	Procaine (2 g/24 h, continuous i.v.)	96 h	VAS (0–100); baseline, and twice daily	Pentazocine	VAS (0–100); total amount of pentazocine
Peiró et al. (43)	16	<12 h	*Included:* Not specified; *Excluded:* Advanced comorbidities, contradictions to anaesthetics	Morphine (1%, 10 mg/4 h, s.c.)	Metamizole (2 g/8 h, i.v. for 3 min)	48 h	VAS (0–100); baseline and every 4 h	Pethidine	Pain free within 24 h (<15 mm VAS)
Layer et al. (36)	44	NR	*Included:* Not specified; *Excluded:* Advanced comorbidities, pregnancy, contradictions to anaesthetics	Procaine (2 g/24 h, i.v.)	Placebo	72 h or until pain free	VAS (0–100); daily and after analgesic given	Metamizole or buprenorphine	Final change of pain intensity (delta VAS 72 h); need for rescue analgesia; response rate
Sadowski et al. (37)	35	NR	*Included:* Ranson ≥ 2 and/or CRP > 100 mg/l and/or necrotising AP; *Excluded:* Contradictions to anaesthetics	Bupivacaine (0.1%, PCEA) + Fentanyl (2 μg/ml, PCEA)	Fentanyl (10 μg/ml, PCIA)	72–120 h	VAS (0–10); every 8 h	Fentanyl PCIA	VAS (0–10); pancreatic blood perfusion
Gülen et al. (38)	90	<24 h	*Included:* VAS > 40/100 mm *Excluded:* Taken NSAID <24 h, advanced comorbidities, trauma, contradictions to anaesthetics	Tramadol (1 mg/kg in 100 ml saline, i.v., for 4–5 min)	Paracetamol (1,000 mg, i.v., for 4–5 min) or dexketoprofen (50 mg/kg, i.v., for 4–5 min)	0.5 h	VAS (0–100); baseline and 30 min after drug given	Morphine	Pain relief at 30 min; need for rescue analgesia
Mahapatra et al. (42)	50	<7 days	*Included:* Organ failure grade <2; *Excluded:* Comorbid coronary artery disease, altered sensorium, serum creatinine ≥ 1.5 mg/dl, paralytic ileus, chronic pancreatitis	Pentazocine (30 mg, Q8h, i.v., for 24 h)	Diclofenac (75 mg, Q8h, i.v., for 24 h)	40 h	NR	Fentanyl PCIA	Dose of rescue analgesic; pain free period; total number of demands of the rescue analgesic
Huang et al. (35)	188	<48 h	*Included:* APACHE II ≥ 8; *Excluded:* AP due to trauma; drug allergy	Conventional treatment	Parecoxib (40 mg per day, p.o., for 3 days) and celecoxib (200 mg, twice daily, p.o., for 7 days)	72 h	VAS (0–10); baseline and every 4 h during the first 3 days	(50–100 mg, i.m.)	VAS (0–10)
Kumar et al. (41)	41	<72 h	*Included:* Not specified; *Excluded:* Advanced comorbidities, pregnancy, contradictions to anaesthetics, lack of informed consent	Diclofenac (1 mg/kg, i.v., over 5 min twice daily)	Tramadol (1 mg/kg, i.v., over 5 min twice daily)	168 h	VAS (0–100); assessed 1, 3, 6, 12 and 24 h after drug given, then every 6 h	Morphine (0.06 mg/kg) after the first 1 h then (0.03 mg/kg) after another 30 min	VAS after 1 h of drug administration and need for rescue analgesia

*NR, not reported; i.m., intramuscular; VAS, visual analogue scale; i.v., intravenous; TTS, transdermal therapeutic system; CRP, C-reactive protein; AP, acute pancreatitis; PCEA, patient-controlled epidural anaesthesia; PCIA, patient-controlled intravenous analgesia; NSAID, non-steroidal anti-inflammatory drug; APACHE II, Acute Physiology, Age and Chronic Health Evaluation II*.

In terms of pharmacological interventions, 4 studies compared analgesics with controls [3 studies with placebo, and one study (35) underwent the same treatments with intervention group without using tested analgesics], 6 studies compared opioids with non-opioids, one study (33) compared opioid with opioid, and one study (37) compared patient-controlled epidural analgesia (PCEA) with patient-controlled intravenous analgesia (PCIA). Rescue analgesics (the primary outcome) in all studies were opioids (4 pethidine, 2 opiates, 2 fentanyl, 2 morphine, 1 pentazocine, and 1 buprenorphine) and 1 study (36) also used metamizole as an alternative to buprenorphine.

### Results for Comparative Data That Cannot Be Quantitively Synthesised

#### Opioid vs. Opioid

Blamey et al. (33) compared the use of opioid (buprenorphine) with another opioid (pethidine) mostly in patients with mild AP (29/32, 90.6%), reporting no significant difference (both *p* ≥ 0.54) in the need for rescue analgesia and adverse events between these two opioids.

#### PCEA vs. PICA

Sadowski et al. (37) compared the PCEA (bupivacaine and fentanyl) with PCIA (fentanyl) in patients with predicted severe AP. While the authors found a decrease in absolute VAS value on the day of PCEA implementation vs. the PCIA regimen (1.6 ± 1.8 vs. 3.5 ± 2.2, *p* = 0.020) and day 10 (0.2 ± 0.4 vs. 2.33 ± 2.3, *p* = 0.034) from respective baselines (6.6 ± 3.4 vs. 7.31 ± 3.4, *p* = 0.572). There were no changes in Δ-VAS from randomisation at all designated time points (all *p* ≥ 0.13; data not shown). The PCEA regimen significantly improved the perfusion of the pancreas compared with the PCIA regimen (13/30, 43% vs. 2/27, 7%, *p* = 0.0025).

### Results of Meta-Analysis

**Figure 2:**
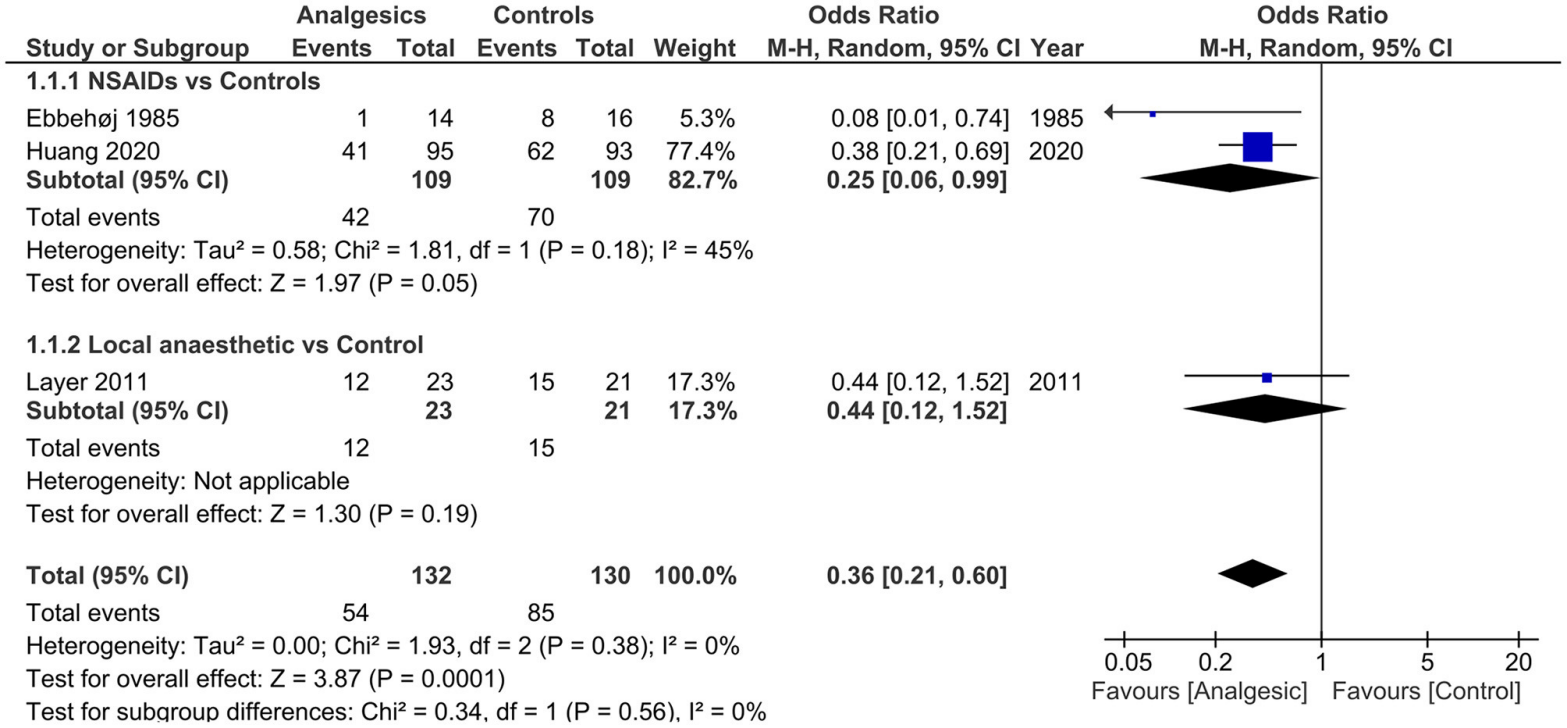
Forest plots of need for rescue analgesia in analgesics vs. controls. M-H, Mantel-Haenszel; CI, confidence interval; NSAIDs, non-steroidal anti-inflammatory drugs.

**Figure 3:**
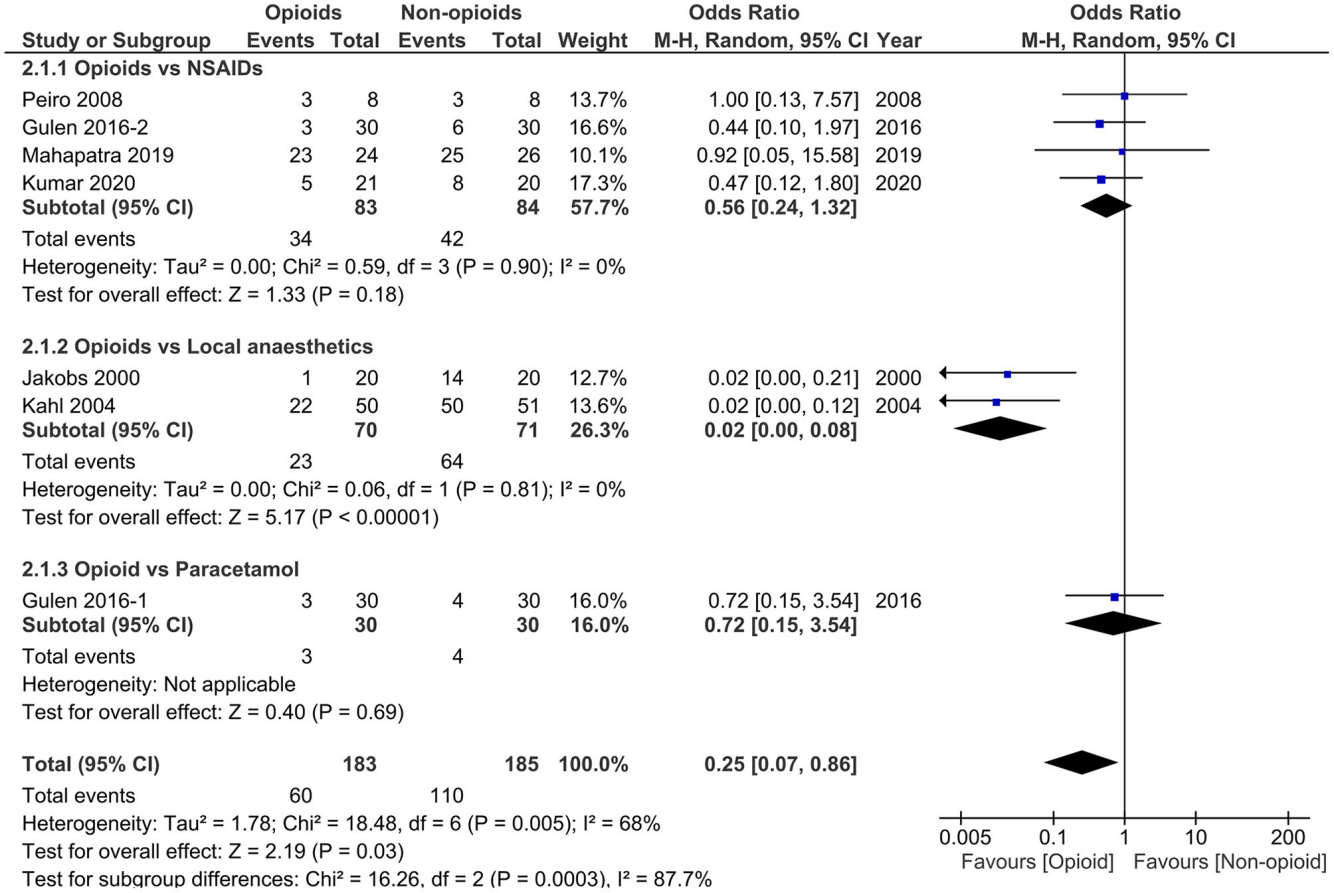
Forest plots of need for rescue analgesia in opioids vs. non-opioids. M-H, Mantel-Haenszel; CI, confidence interval; NSAIDs, non-steroidal anti-inflammatory drugs.

Data from 4 studies (34–36, 44) comparing analgesics with controls (placebo or conventional treatment) and 6 studies (38–43) comparing opioids with non-opioids were quantitatively synthesised for analysis. Results of the meta-analysis of primary outcome measure are presented in Figures 2, 3. All outcome measures are summarised in Table 4. Results of subgroup analysis are presented in Table 5.

**Table 4:** Results of meta-analyses.

**Outcomes of interest**		**No. of patients**		**Effect estimate**		**herogeneity**	
	**No. of studies**	**Experimental**	**Control**	**WMD/OR (95% CI)**	***P*** **-value**	***I^2^*** **(%)**	***P*** **-value**
**Analgesics vs. Controls**							
**Pain related outcome**							
Need for rescue analgesia	3	132	130	0.36 (0.21, 0.60)	0.0001	0	0.38
Initial VAS (0–100)	4	147	147	0.93 (-8.15, 10.01)	0.84	83	0.0006
Δ-VAS at 24 h	2	118	114	18.46 (0.84, 36.07)	0.04	94	<0.0001
Δ-VAS on 3–7d	3	132	130	11.57 (0.87, 22.28)	0.03	98	<0.00001
**AP related outcome**							
Any complication	2	118	114	0.36 (0.11, 1.16)	0.09	58	0.12
Mortality	2	109	109	1.06 (0.22, 5.19)	0.94	0	0.45
Length of hospital stay (d)	2	110	110	−4.76 (-11.46, 1.95)	0.16	99	<0.00001
**Opioids vs. Non-opioids**							
**Pain related outcome**							
Need for rescue analgesia	6	153	185	0.25 (0.07, 0.86)	0.03	68	0.005
Initial VAS (0–100)	5	129	159	3.28 (-0.07, 6.63)	0.06	34	0.18
Δ-VAS within 24 h	4	109	139	6.06 (-18.10, 30.22)	0.62	98	<0.00001
**AP related outcome**							
Any complication	5	123	125	1.16 (0.61, 2.20)	0.66	0	0.41
Local complication	2	32	34	0.62 (0.05, 7.80)	0.71	48	0.16
Organ failure	4	73	74	1.74 (0.41, 7.36)	0.45	39	0.19
Mortality	3	65	66	0.69 (0.11, 4.52)	0.70	0	0.56
Length of hospital stay (d)	2	44	46	−3.03 (-7.34, 1.28)	0.17	74	0.05
**Drug safety outcome**							
Adverse events	5	132	165	1.52 (0.55, 4.21)	0.42	25	0.25

*WMD, weighted mean difference; OR, odds ratio; CI, confidence interval; NSAID, non-steroidal anti-inflammatory drug; VAS, visual analogue scale; AP, acute pancreatitis*.

**Table 5:** Subgroup analysis of need for rescue analgesia.

**Outcomes of interest**		**No. of patients**		**Effect estimate**		**Heterogeneity**	
	**No. of studies**	**Experimental**	**Control**	**WMD/OR (95% CI)**	***P*** **-value**	***I^2^*** **(%)**	***P*** **-value**
**Analgesics vs. Controls**							
*NSAIDs* vs. *Controls*	2	109	109	0.25 (0.06, 0.99)	0.05	45	0.18
**Opioids vs. Non-opioids**							
*Opioids vs. NSAIDs*	4	83	84	0.56 (0.24, 1.32)	0.18	0	0.90
*Opioids vs. Local anaesthetics*	2	70	71	0.02 (0.00, 0.08)	<0.00001	0	0.81

*WMD, weighted mean difference; OR, odds ratio; CI, confidence interval; NSAID, non-steroidal anti-inflammatory drug*.

#### Analgesics vs. Controls

Three (34–36) (*n* = 262) out of the 4 included studies (34–36, 44) reported the primary outcome. The pooled results demonstrated that the use of analgesics significantly reduced the need for rescue analgesia (OR 0.36, 95% CI.0.21 to 0.60, *p* = 0.0001) without heterogeneity (*I*^2^ = 0) vs. controls.

With comparable initial VAS scores in both groups (*p* = 0.84), the analgesic group significantly improved pain intensity (Δ-VAS) within 24 h [2 studies (35, 36), *n* = 232; WMD 18.46, 95% CI.0.84 to 36.07, *p* = 0.04] and 3 to 7 days [3 studies (34–36), *n* = 262; WMD 11.57, 95% CI 0.87 to 22.28, *p* = 0.03], albeit with high heterogeneity (both *I*^2^ ≥ 94%). There were no significant differences in terms of any complication, mortality, length of hospital stay, and adverse events (all *p* ≥ 0.09).

Subgroup analysis of NSAIDs vs. controls still resulted in reduced need for rescue analgesia [2 studies (34, 35), *n* = 218; OR 0.25, 95% CI 0.06 to 0.99, *p* = 0.05] with low study of heterogeneity (45%).

#### Opioids vs. Non-opioids

All 6 studies (38–43) (*n* = 338) reported the primary outcome [1 (42) by personal communication]. The pooled results indicated that opioids significantly reduced the need for rescue analgesia compared with non-opioids (OR 0.25, 95% CI 0.07 to 0.86, *p* = 0.03) with high study heterogeneity (*I*^2^ = 68%). The initial pain score was slightly higher in opioids group than non-opioids group [5 studies (38–41, 43), *n* = 288; WMD 3.28, 95% CI−0.07 to 6.63, *p* = 0.06] and pooled data demonstrated no significant difference regarding the Δ-VAS within 24 h in these two groups [4 studies (38, 40, 41, 43), *n* = 248; WMD 6.06, 95% CI-18.10 to 30.22, *p* = 0.62] with high heterogeneity (*I*^2^ = 98%). The other clinical outcomes were not significantly different between these two groups (all *p* ≥ 0.17).

In subgroup analysis, when restricting studies to those comparing opioids with NSAIDs, no significant difference in the need for rescue analgesia was observed [4 studies (38, 41–43), *n* = 167; OR 0.56, 95% CI 0.24 to 1.32, *p* = 0.18]. However, the primary results remained significant when comparing opioids with systemic use of local anaesthetics [2 studies (39, 40), *n* = 141; OR 0.02, 95% CI 0.00 to 0.08, *p* < 0.00001]. There was no study heterogeneity in both subgroup analyses (both *I*^2^ = 0). These results demonstrated that the superiority of opioids in opioids vs. non-opioids analysis was driven by the results of the opioids vs. local anaesthetics.

### Sensitivity Analysis

The sensitivity analysis could only be performed for opioids vs. non-opioids (Supplementary Table 3). The primary results of the need for rescue analgesia became insignificant by only analysing studies of high quality (38, 41, 42) (3 studies, *n* = 181; OR 0.55, 95% CI 0.24 to 1.23, *p* = 0.14) with improved heterogeneity (68% to 0). The results were not affected by only including studies with sample size ≥ 40 (38–42), Western population (39, 40, 43), or by studies mainly including patients with mild AP and these had similar study heterogeneity.

### GRADE Assessment

The GRADE could only be applied to the studies of opioids vs. non-opioids (Table 6). Moderate quality of evidence was rated for the need for rescue analgesia. Low quality of evidence was rated for other outcomes assessed including Δ-VAS within 24 h, any complication, length of hospital stay, and adverse events.

**Table 6:** Grading of Recommendations, Assessment Development and Evaluations (GRADE) evidence profile and summary of findings: Opioids vs. Non-opioids.

**Certainty assessment**							**No. of patients**		**Effect**		**Certainty**
**Outcomes**	**No. of studies**	**Risk of bias**	**Inconsistency**	**Indirectness**	**Imprecision**	**Other considerations**	**Opioid**	**Non-opioid**	**Relative (95% CI)**	**Absolute (95% CI)**	
**Need for rescue analgesia**	6 RCTs	serious^b^, ^c^, ^e^	serious^d^	not serious	not serious	strong association	60/183 (32.8%)	110/185 (59.5%)	OR 0.25 (0.07 to 0.86)	326 fewer per 1,000 (from 501 fewer to 37 fewer)	⊕⊕⊕◯ MODERATE
**ΔVAS within 24 h (follow up: 0.5 h to 24 h)**	4 RCTs	serious^b^, ^c^	serious ^a^, ^d^	not serious	not serious	none	109	139	-	MD 6.06 higher (18.10 lower to 30.22 higher)	⊕⊕◯◯ LOW
**Any complication**	5 RCTs	serious^b^, ^c^, ^e^	serious ^d^, ^f^	not serious	not serious	none	59/123 (48.0%)	59/125 (47.2%)	OR 1.16 (0.61 to 2.20)	37 more per 1,000 (from 119 fewer to 191 more)	⊕⊕◯◯ LOW
**Length of hospital stay**	2 RCTs	serious^e^	serious^d^	not serious	not serious	none	42	46	-	MD 3.03 lower (7.34 lower to 1.2 higher)	⊕⊕◯◯ LOW
**Adverse events**	5 RCTs	serious^b^, ^c^, ^e^	serious^d^	not serious	not serious	none	18/162 (11.1%)	14/165 (8.5%)	OR 1.52 (0.55 to 4.21)	37 more per 1,000 (from 35 fewer to 191 more)	⊕⊕◯◯ LOW

*CI, confidence interval; RCT, randomised controlled trial; VAS, visual analogue scale; MD, mean difference; OR, odds ratio.*

a *Variation of time points for pain scoring.*

b *Unblinded of study by Kahl et al. (40).*

c *Unblinded of study by Peiro et al. (43).*

d *Variation of dose and approach for drug administration.*

e *Unblinded of study by Jakobs et al. (39).*

f *Definitions of complications of acute pancreatitis were not consistent across different studies. GRADE levels of quality of evidence: High, further research is very unlikely to change our confidence in the estimate of effect; Moderate, further research is likely to have an important impact on our confidence in the estimate of effect and may change the estimate; Low, further research is very likely to have an important impact on our confidence in the estimate of effect and is likely to change the estimate; Very low, any estimate of effect is very uncertain*.

### Publication Bias

There was no convincing evidence of publication bias for the primary outcome measure in opioids vs. non-opioids (Begg-Mazumdar and Egger: *p* > 0.1) by funnel plot analysis (Supplementary Figure 1).

## Discussion

This systematic review and meta-analysis included 12 RCTs investigating analgesics for pain relief in AP and comprised a total of 699 patients, of whom 83% were mild cases. It was found that the use of analgesics was associated with reduced need for rescue analgesia and with pain score compared with controls. Unlike the previous meta-analysis (22) showing opioids were equivalent to non-opioids in decreasing the need for rescue analgesia, this meta-analysis found that opioids were superior to non-opioids in regards to the need for rescue analgesia but without significant differences in pain score, other clinical outcomes, and adverse events. It was found that NSAIDs and opioids were equally effective in decreasing the need for rescue analgesia in patients with AP, which was not the case for the systemic administration of local anaesthetics. The lack of heterogeneity in these two subgroups analyses makes the conclusion more convincing.

Of the 4 RCTs that investigated the effects of analgesics in comparison with controls, 2 reported that NSAIDs reduced the need for rescue analgesia. Ebbehoj et al. (34) showed that indomethacin was effective for pain relief. In a most recent study, Huang et al. (35) demonstrated that parecoxib plus celecoxib (selective COX-2 inhibitors) was associated with significantly less meperidine injections, reduced pain score, systemic and local complications, length of hospital stay, and cost without evidence of adverse events. Indeed, NSAIDs (i.e., celecoxib) are strongly recommended by the EARS Society as a component of a multimodal analgesic strategy and have been shown to reduce PCIA morphine consumption after major surgery in patients without contraindications (45, 46). Overall, our findings support the use of NSAIDs (in the absence of acute kidney injury) for pain relief in AP which is not a feature in previous AP guidelines.

Compared with the Cochrane database review conducted by Basurto et al. (22) in 2013 which included 5 RCTs with 227 participants, the current systematic review included 12 RCTs with 699 participants. For opioids vs. non-opioids, while Basurto et al. (22) did not show significance (3 studies with 162 patients, OR 0.41, 95% CI 0.14 to 1.19, *p* = 0.10), our results of an additional 3 RCTs favoured opioids over non-opioids (6 studies with 338 patients; OR 0.25, 95% CI 0.07 to 0.86, *p* = 0.03) in reducing the need for rescue analgesia. Subgroup analysis of 2 studies comparing opioids with procaine did not change the result of the primary outcome (OR 0.02, 95% CI 0.00 to 0.08, *p* < 0.00001), indicating opioids are a more effective analgesic than the systemic administration of procaine. The rationale for using procaine is because of its local analgesic effect and, possibly, its inhibitory effect on phospholipase A2 catalytic activity, which is an important enzyme involved in the early pathogenesis of AP (47). Therefore, systemic administration of local anaesthetics, mainly procaine, was already recommended as front-line analgesic in AP by consensus conferences in several European countries two decades ago (48, 49). Our results suggested that systemic use of local anaesthetics is not supported and, thus, its role in pain management in AP needs to be defined.

Subgroup analysis of 4 studies between opioids and NSAIDs revealed there was no significant difference in the need for additional analgesic (OR 0.56, 95% CI 0.24 to 1.32, *p* = 0.18) without heterogeneity (*I*^2^ = 0), Δ-VAS, other clinical outcomes, and adverse events. Recently, a retrospective study conducted by Kim et al. (50) showed that opioid was associated with an increased risk of developing AP compared to NSAIDs (OR 2.64, 95% CI 1.54 to 4.52). It is reported that opioids can potentially cause AP in patients with a history of cholecystectomy due to its adverse effect of sphincter of Oddi constriction (50, 51). There are other effects of opioids including ileus, dysbiosis, opioid hyperalgesia, and others (22, 52). However, opioids have been overused for pain management in AP especially in North America where 92.5% of patients with AP received opioids for impatient pain management and 64.3% have been given opioids at discharge (53). On the other hand, the over prescription or prescription of opioids without adequate supervision has led to an alarming rise of opioid overdose-associated deaths (54). From 2013 to 2019, the synthetic opioid-associated death rate increased more than 10 folds, from 1.0 to 11.4 per 100,000 age-adjusted population in the United States (55). Therefore, based on the available evidence, it appears that NSAIDs are preferred over opioids as first line analgesia in patients with AP.

Epidural analgesia is an essential component of most enhanced recovery after surgery (ERAS) pathways and is commonly used in major abdominal surgery, because it is associated with superior pain control (46). However, epidural analgesia is rarely used in patients with AP. Sasabuchi et al. (56) investigated 44,146 patients with AP in Japan between 2010 and 2013 and found that only 0.7% of patients received epidural analgesia for pain management. A large propensity score-matched retrospective observational study (57) of 1,003 patients conducted in 2018 demonstrated that the mortality of critically ill patients with AP who received epidural analgesia was significantly lower than those who did not. In our study, there was only one RCT focusing on PCEA vs. PCIA (37). It was clear that PCEA markedly improved pancreatic arterial perfusion over PCIA, and this finding is consistent with studies from experimental AP models (26) and with patients who are critically ill with AP (37). Meanwhile, a multi-centre RCT (58) is underway to elucidate the benefits of PCEA among critically ill patients with AP. PCEA is not currently recommended for patients with in mild and moderately severe AP because of potential adverse effects including catheter placement-related hypotension and epidural abscess, although at a relatively low incidence. More studies evaluating the safety and efficacy of epidural analgesia in patients with severe AP are warranted.

In our study, we did not include studies assessing the analgesic effect of acupuncture on patients with AP as we focused on the pharmacological intervention. A systematic review and meta-analysis (59) have demonstrated that acupuncture was associated with significantly reduced abdominal pain (mean difference −0.87, 95% CI −1.01 to −0.73, *p* < 0.00001), improved gastrointestinal function, accelerated time of resuming to diets, and shortened the length of hospital stay without noticeable adverse events. However, most of these studies are published in Chinese and more rigorously designed RCTs are needed to confirm these findings.

It is striking that there is not more level 1 evidence available to guide decision-making with optimised analgesic protocols in patients with AP. The evidence available from the 12 RCTs is limited by the quality and heterogeneity of included studies as shown by our sensitivity analysis. In order to minimise the impact of heterogeneity, the random-effects model was adopted in the quantitative analysis for all outcomes. The unblinding characteristic of some studies may influence the patient-reported outcomes including the scoring of pain. Moreover, the variation in drug administration route and dosages impacted the study outcomes and this is reflected in the low GRADE levels. For example, in opioids vs. non-opioids, Peiró et al. (43) utilised smaller doses of morphine subcutaneously while other studies administered greater doses of opioids by intravenous approach. This might also explain the differences between studies. Other aspects that impact the trial design and outcomes are differences in the timing of pain onset in relation to hospital admission or recruitment, admission pain intensity, and predicted severity. We chose the primary outcome to be “the need for rescue analgesia” as data were not available on opioid equivalent for the rescue analgesic in most studies. For future trials, there is also the need for standardised reporting of pain outcomes and inclusion the identification of objective biomarkers (electrophysiology, bioassay, omics, imaging, and behaviour) (54).

The important finding from this study is that NSAIDs are as effective as opioids and opioids are more effective than systemic local anaesthetics on reducing the need for rescue analgesia in patients with mild AP. Epidural anaesthesia shows promise but will need further trials in patients with predicted severe AP before it can be recommended or adopted.

## Data Availability Statement

The original contributions presented in the study are included in the article/Supplementary Materials, further inquiries can be directed to the corresponding authors.

## Author Contributions

WC, FL, and YW are joint first authors of this article, undertook the statistical analysis, and interpreted data. WH conceived and designed the study. WC, FL, YW, and CH undertook the systematic literature search, acquisition of data, quality cheque, and risk of bias assessment. MP contributed to the design of statistical analysis. WC, FL, YW, and WH drafted the manuscript. WH and QX obtained funding and supervised the study. VS and RS had important intellectual input. All authors have approved the final version of manuscript before submission.

## Funding

This project is supported by the National Nature Science Foundation of China (No. 82104598, CH; No. 81973632, WH). NZ-China Strategic Research Alliance 2016 Award (China: 2016YFE0101800, QX and WH). WC is funded by China Scholarship Council (No. 201806240274) and West China Hospital-Liverpool Clinician Scientist Development Award.

## Conflict of Interest

The authors declare that the research was conducted in the absence of any commercial or financial relationships that could be construed as a potential conflict of interest.

## Publisher's Note

All claims expressed in this article are solely those of the authors and do not necessarily represent those of their affiliated organizations, or those of the publisher, the editors and the reviewers. Any product that may be evaluated in this article, or claim that may be made by its manufacturer, is not guaranteed or endorsed by the publisher.
