# Supplementary material for: Pain Management in Acute Pancreatitis: A Systematic Review and Meta-Analysis of Randomised Controlled Trials
Source: Front Med (Lausanne). 2021 Dec 17;8:782151. doi: 10.3389/fmed.2021.782151 (PMC8718672; doi:10.3389/fmed.2021.782151)
Supplement: Supplementary file 2 [file Data_Sheet_1.docx]

**Supporting information**

**Appendix S1 Detailed search strategy**

1. exp Pancreatitis/
2. exp Acute disease/
3. 1 and 2
4. Acute adj3 pancreatitis/
5. 3 or 4
6. Exp Randomized Controlled Trial/
7. Exp Random Allocation/
8. Exp Double-Blind Method/
9. Exp Single-Blind Method/
10. Exp Clinical Trial/
11. Clinical Trials as Topic
12. clinical trial, phase i.pt
13. clinical trial, phase ii.pt
14. clinical trial, phase iii.pt
15. clinical trial, phase iv.pt
16. ((single or double or tripl$) adj (blind$ or mask$)).tw
17. placebo.mp.
18. (random$ adj2 allocated).tw
19. or/6-18
20. exp Analgesics, Opioid/
21. (opioid$ or opiate$ or narcotic$).mp.
22. exp Morphine/
23. exp Opium/
24. exp Hydromorphone/
25. exp Codeine/
26. ﻿exp Oxycodone/
27. ﻿exp Meperidine/
28. ﻿exp Fentanyl/
29. ﻿exp Dextromoramide/
30. ﻿exp Methadone/
31. ﻿exp Pentazocine/
32. ﻿exp Phenazocine/
33. ﻿exp Buprenorphine/
34. ﻿exp Etorphine/
35. ﻿exp Morphinans/
36. ﻿exp Butorphanol/
37. ﻿ exp Tilidine/
38. ﻿exp Tramadol/
39. ﻿exp Meptazinol/
40. or/20-39
41. exp Anti-Inflammatory Agents, Non-Steroidal/
42. exp Ibuprofen/
43. exp Naproxen/
44. exp Diclofenac/
45. exp Celecoxib/
46. exp Mefenamic Acid/
47. exp Etoricoxib/
48. exp Indomethacin/
49. exp Acetaminophen/
50. paracetamol.mp.
51. or/41-50
52. exp Anesthetics, Local/
53. exp Procaine/
54. exp Lidocaine/
55. exp Bupivacaine/
56. exp Ropivacaine/
57. exp Mepivacaine/
58. exp Benzocaine/
59. exp Cocaine/
60. or/52-59
61. exp Anesthetics, Epidural/
62. 40 or 51 or 60 or 61
63. 5 and 19 and 62

**Table S1**. Quality assessment of included studies by the Jadad Score

|  | Randomisation | Double blinding | Drop-out or withdrawal | Allocation concealment | Jadad score |
| --- | --- | --- | --- | --- | --- |
| Blamey *et al.* | 1 | 1 | 0 | 1 | 3 |
| Ebbehoj *et al*. | 0 | 2 | 0 | 0 | 2 |
| Jakobs *et al*. | 1 | 0 | 1 | 0 | 2 |
| Stevens *et al*. | 1 | 2 | 1 | 1 | 5 |
| Kahl *et al*. | 1 | 0 | 1 | 0 | 2 |
| Peiró *et al*. | 1 | 0 | 0 | 1 | 2 |
| Layer *et al*. | 1 | 2 | 1 | 1 | 5 |
| Sadowski *et al*. | 1 | 0 | 1 | 0 | 2 |
| Gülen *et al.* | 1 | 1 | 1 | 1 | 4 |
| Mahapatra *et al.* | 1 | 1 | 1 | 1 | 4 |
| Kumar *et al.* | 1 | 2 | 1 | 1 | 5 |
| Huang *et al.* | 1 | 0 | 1 | 1 | 3 |

**Table S2.** Baseline characteristics of included patients

| **Study** | **Patient screened (analysed)** | **Groups** | **No. of**  **patients** | **Age**  **(year)** | **Gender**  **(Male/Female)** | **Weight**  **or BMI** | **Aetiology** | |  |  | **Severity** |  |
| --- | --- | --- | --- | --- | --- | --- | --- | --- | --- | --- | --- | --- |
|  |  |  |  |  |  |  | **Biliary** | **Alcohol** | | **Others** |  |  |
| Blamey *et al*. | 32 (32) | Pethidine | 15 | NR | NR | NR | NR | NR | | NR | Prognostic factor scoring | 2 SAP |
|  |  | Buprenorphine | 17 |  |  |  |  |  | |  |  | 1 SAP |
| Ebbehoj *et al*. | 30 (30) | Indomethacin | 14 | 27-83 | 9/5 | NR | 3 | 6 | | 5 | Ranson criteria ≥ 3 | n = 3 |
|  |  | Placebo | 16 | 27-86 | 11/5 |  | 5 | 8 | | 3 |  | n = 4 |
| Jakobs *et al*. | 40 (40) | Buprenorphine | 20 | 26-76 | 12/8 | 55-93 kg | 5 | 9 | | 5 | APACHE II, median (range) | 5 (0-16) |
|  |  | Procaine | 20 | 23-72 | 11/9 | 48-88 kg | 3 | 14 | | 3 |  | 3 (0-18) |
| Stevens *et al*. | 42 (32) | Fentanyl | 15 | 26-47 | 18/14 | NR | NR | NR | | NR | NR |  |
|  |  | Placebo | 17 | 26-47 |  | NR | NR | NR | | NR |  |  |
| Kahl *et al*. | 107 (101) | Pentazocine | 50 | 43 ± 11 | 38/12 | 77 ± 17 kg | 14 | 36 | | 0 | CT severity index and APACHE II | 0 SAP |
|  |  | Procaine | 51 | 47 ± 14 | 34/17 | 73 ± 14 kg | 16 | 35 | | 0 |  | 1 SAP |
| Peiró *et al*. | 16 (16) | Morphine | 8 | 55.1 ± 18.8 | 5/3 | NR | 2 | 3 | | 3 | APACHE II > 6 | n = 2 |
|  |  | Metamizole | 8 | 54.4 ± 13.5 | 3/5 | NR | 6 | 1 | | 1 |  | n = 1 |
| Layer *et al*. | 46 (44) | Procaine hydrochloride | 23 | > 55 (12) | 16/7 | >25.5 kg/m^2^ (9) | 6 | 11 | | 6 | NR | Most patients had MAP |
|  |  | Placebo | 21 | >55 (10) | 10/11 | >25.5 kg/m^2^ (12) | 9 | 7 | | 5 |  |  |
| Sadowski et al. | 49 (35) | Bupivacaine + Fentanyl | 13 | 66.1 (11.7) | 7/6 | 27.8 (6.1) kg/m^2^ | 7 | 3 | | 3 | Predicted SAP^a^ | ﻿n = 13 |
|  |  | Fentanyl | 22 | 57.4 (18) | 12/10 | 29.2 (7.1) kg/m^2^ | 13 | 6 | | 3 |  | ﻿n = 22 |
| Gülen et al. | 116 (90) | Tramadol | 30 | 53.5 ± 13.3 | 53/37 | NR | 66 | 19 | | 5 | NR |  |
|  |  | Paracetamol | 30 |  |  |  |  |  |  |  |  |  |
|  |  | Dexketoprofen | 30 |  |  |  |  |  |  |  |  |  |
| Mahapatra et al. | 86 (50) | Pentazocine | 24 | 41.3 (11.8) | 12/12 | NR | 6 | 3 | | 15 | RAC | 24 MAP |
|  |  | Diclofenac | 26 | 35.1 (11.8) | 12/14 | NR | 7 | 5 | | 13 |  | 26 MAP |
| Kumar et al. | 46 (41) | Diclofenac | 20 | 46.8 ± 5.6 | 14/6 | NR | 7 | 12 | | 1 | BISAP, median (IQR) | 1 (1.5) |
|  |  | Tramadol | 21 | 48 ± 12.8 | 13/8 | NR | 13 | 4 | | 3 |  | 1.5 (1) |
| Huang et al. | 190 (188) | Conventional treatment (C) | 93 | 44.83 ± 10.39 | 53/40 | 25.43 ± 2.16 kg/m^2^ | 39 | 20 | | 34 | RAC | 37 SAP |
|  |  | C + parecoxib  celecoxib  (NSAIDs) | 95 | 46.72 ± 11.18 | 56/39 | 25.75 ± 1.89 kg/m^2^ | 40 (42.11%) | 21 (22.10%) | | 34 (35.79%) |  | 20 SAP |

BMI, body mass index; NR, not reported; SAP, severe acute pancreatitis; APACHE II, ﻿Acute Physiology and Chronic Health Evaluation II; CT, ﻿computerised tomography; SD, standard deviation; RAC, revised Atlanta classification; MAP, mild acute pancreatitis; BISAP, bedside index for severity in acute pancreatitis; SIRS, Systemic Inflammatory Response Syndrome.

^a^ ﻿Ranson score ≥ 2, C-reactive protein > 100, or necrosis on computed tomography.

**Table S3.** Results of sensitivity analysis: Opioids versus Non-opioids

| **Outcomes of interest** |  | **No. of patients** | | **Effect estimate** | | **Heterogeneity** | |
| --- | --- | --- | --- | --- | --- | --- | --- |
|  | **No. of studies** | **Opioids** | **Non-opioids** | **OR (95% CI)** | ***P* value** | ***I^2^* (%)** | ***P* value** |
| **Need for rescue analgesia** |  |  |  |  |  |  |  |
| High quality RCTs | 3 | 75 | 106 | 0.55 (0.24, 1.23) | 0.14 | 0 | 0.95 |
| *Sample size ≥ 40* | 5 | 145 | 177 | 0.20 (0.05, 0.79) | **0.02** | 70 | 0.005 |
| *Western population* | 3 | 78 | 79 | 0.07 (0.00, 1.05) | **0.05** | 80 | 0.008 |
| *Mainly mild AP* | 5 | 123 | 125 | 0.17 (0.03, 1.02) | **0.05** | 75 | 0.003 |

OR, odds ratio; CI, confidence interval; RCT, randomised clinical trials, AP, acute pancreatitis.

**Fig. S1** Publication bias

Funnel plot of publication bias for need for rescue analgesia in opioid vs non-opioid.

Bias indicators

Begg-Mazumdar: Kendall's tau = -0.047619, P = 0.7726 (low power).

Egger: bias = -2.337829 (95% CI = -9.716997 to 5.041339), P = 0.4524.
